# Sodium Butyrate Attenuates Diabetic Kidney Disease Partially via Histone Butyrylation Modification

**DOI:** 10.1155/2022/7643322

**Published:** 2022-07-20

**Authors:** Tingting Zhou, Huiwen Xu, Xi Cheng, Yanqiu He, Qian Ren, Dongzhe Li, Yumei Xie, Chenlin Gao, Yuanyuan Zhang, Xiaodong Sun, Yong Xu, Wei Huang

**Affiliations:** ^1^Department of Endocrinology and Metabolism, Metabolic Vascular Diseases Key Laboratory of Sichuan Province, The Affiliated Hospital of Southwest Medical University, Luzhou, Sichuan 646000, China; ^2^Sichuan Clinical Research Centre for Nephropathy, Luzhou, Sichuan 646000, China; ^3^Cardiovascular and Metabolic Diseases Key Laboratory of Luzhou, Sichuan 646000, China; ^4^Department of Endocrinology and Metabolism, People's Hospital of Deyang City, Sichuan 618000, China; ^5^China School of Pharmacy, Sichuan University, Chengdu, Sichuan 610041, China; ^6^West China School of Basic Medical Sciences & Forensic Medicine, Sichuan University, Chengdu, Sichuan 610041, China

## Abstract

Inflammation and fibrosis are the important pathophysiologic processes in diabetic kidney disease (DKD), which is induced by epigenetics, especially histone posttranslational modification (HPTMs). Recent reports highlighted that butyrate, one of the short-chain fatty acids (SCFAs) primarily originated from the fermentation of dietary fiber in the gut, attenuates inflammation and fibrosis in the prevention and treatment of DKD; however, the molecular mechanisms are still unclear. Histone lysine butyrylation (Kbu), a novel histone modification marker induced by butyrate, has been found to be involved in the regulation of pathophysiological processes. To reveal the mechanisms of butyrate-induced histone (Kbu), in the prevention and treatment of DKD, both DKD models *in vivo* and *in vitro* were treated with sodium butyrate (NaB). Our results confirmed that exogenous NaB improved the disorder of glucose and lipid metabolism, prevented proteinuria and renal failure, and inhibited renal inflammation and fibrosis. Meanwhile, NaB also induced histone Kbu and H3K9 butyrylation (H3K9bu) *in vivo* and *in vitro*; however, inhibition of histone Kbu with the histone modification enzyme p300 inhibitor A485 reversed the anti-inflammatory and anti-fibrosis effects of NaB. In conclusion, our data reveal that NaB antagonizes renal inflammatory and fibrosis injury and attenuates DKD possibly via histone Kbu, suggesting that butyrate-induced histone Kbu or H3K9bu may be an important molecular mechanism in the pathogenesis and treatment of DKD.

## 1. Introduction

Diabetic kidney disease (DKD), the typical microvascular complication of diabetes mellitus, is the leading cause of end-stage renal disease (ESRD) [1]. The pathogenesis of DKD is the result of an interaction between genetic and environmental factors; up to now, there still lacks effective therapy for DKD [2]. The latest research found that histone posttranslational modifications (HPTMs) regulate the transcriptional activity of inflammation and fibrosis-related genes, such as interleukin-6 (IL-6) and transforming growth factor-*β* (TGF-*β*), by altering the loose or condensed state of chromatin, which is involved in the pathogenesis of DKD [3, 4]. Compared with genetic factors, histone modification, as the “link” between a high glucose environment and DKD, is relatively reversible, so it may be a new breakthrough in the prevention and treatment of DKD [5].

Butyrate is one of the short-chain fatty acids primarily originating from the fermentation of dietary fiber in the gut; however, it can reach the bloodstream and is involved in inflammatory and immune-associated diseases such as inflammatory bowel disease, asthma, arthritis, and other inflammatory diseases [6–8]. Previous studies found that sodium butyrate (NaB) could be a potential therapeutic agent in the prevention and treatment of DKD *in vivo* and *in vitro* [9]. However, the mechanism of NaB ameliorating DKD is unclear; it is speculated that the HPTMs are a possible signaling pathway. Histone lysine butyrylation (Kbu) was firstly identified by mass spectrometry- (MS-) based proteomics in 2007 [10]. At present, butyrate-induced butyryl-CoA via acetyl-CoA synthetase 2 (ACSS2) is considered to be the corresponding substrate donating butyryl, which is essential to the acylation reaction of histone Kbu. The acetyltransferase activity transcriptional coactivator P300/CBP is the common acyltransferase, which transfers butyryl to the histone lysine and rapidly promotes histone Kbu [11]. As a novel HPTM maker, histone Kbu provides a new route for revealing the pharmacological effect of butyrate. However, the relationship between histone Kbu and DKD and the role and mechanism of butyrate in this modification process are still unclear.

In this study, we firstly confirmed the effects of NaB on streptozotocin- (STZ-) induced DKD mouse models *in vivo* and high glucose-induced mouse glomerular mesangial cells (GMCs) *in vitro*; histone Kbu, H3K9 butyrylation (H3K9bu), and DKD-related genes or proteins were detected; finally, we evaluated whether histone Kbu is involved in anti-inflammatory and an-tifibrosis effects of NaB by A485, a block of histone Kbu. Our findings elucidated the potential mechanisms of butyrate protective function in DKD and gave some clues for the potential therapy for DKD.

## 2. Materials and Methods

### 2.1. Animal Model

Eight-week-old male C57BL/6 mice were purchased from the Biotechnology Corporation of Dashuo (Chengdu, China). All animal care and investigation were approved by Southwest Medical University. After 1 week of adaptive feeding, all mice were randomly divided into a control group (NC group, *n* = 8) and a DKD group (*n* = 16). The NC group was given a normal diet until the end of the experiment, while the DKD group was given a high-fat diet (60% calorie fat, Dashuo Biotech, China) for 8 weeks, and then, diabetes was induced by intraperitoneal injection of a single low dose (50 mg/kg) of STZ (Beijing Solebro Technology Co Ltd, China) for 5 days to induce diabetes, followed by continued HFD feeding for an additional 12 weeks. A random blood glucose level ≥ 16.7 mmoL/L for 3 days was confirmed as “diabetic.” DKD mice were randomly divided into 2 groups (*n* = 8/group) of 8 mice each with the same mean initial body weight: [1] the sodium butyrate group (NaB group): based on the previous experimental study of our group [9], DKD mice were treated with sodium butyrate (Sigma-Aldrich, USA) 40 mg/(kg·48 h) intraperitoneally for 12 weeks; [2] the DKD control group (DKD group): DKD mice were injected intraperitoneally with the same volume and frequency of phosphate-buffered saline (PBS). At the same time, the NC group was also injected with an equal volume and frequency of PBS buffer. Body weight and blood glucose were recorded every two weeks.

### 2.2. Biochemical Measurements

Random blood glucose (RBG) and fasting blood glucose (FBG) levels were measured with an Accu-Chek (Roche Diagnostics, Mannheim, Germany). Urine albumin-creatinine ratios (ACR) were assayed according to the manufacturer's procedures outlined in the kit (Andygene, USA). Blood creatinine (crea), urea nitrogen (BUN), triglyceride (TG), total cholesterol (TC), and low-density lipoprotein-cholesterol (LDL-C) levels were analyzed using kits (Nanjing Jiancheng Bioengineering Institute, China) according to the manufacturer's protocols.

### 2.3. Renal Histology

The kidneys were rapidly dissected and fixed in 10% buffered formalin at 4°C overnight. The kidneys were embedded in paraffin and were sectioned at 5 *μ*m thickness on positively charged slides. Sections were stained with hematoxylin and eosin (H&E) and Masson's trichrome staining for light microscopic analysis and morphometry.

### 2.4. Immunohistochemistry Staining

Sections were incubated with the following primary antibodies: anti-PanKbu (mouse polyclonal antibody; 1 : 200 dilution; Hangzhou Jing Jie biological Co., Ltd; China), anti-H3K9bu (mouse polyclonal antibody; 1 : 200 dilution; Hangzhou Jing Jie biological Co., Ltd; China), anti-Fn (rabbit polyclonal antibody; 1 : 200 dilution; Abcam; UK), and anti-P300 (rabbit polyclonal antibody; 1 : 200 dilution; Cell Signaling Technology; USA) overnight at 4°C. After sections were washed with PBS, they were incubated with horseradish peroxidase (HRP) or fluorescein isothiocyanate fluorescent dye-conjugated secondary antibodies (1 : 200 dilution; Beijing Biosynthesis Biotechnology; China) for 2 h at room temperature. For visualizing the signals of immunohistochemistry, sections were treated with peroxidase substrate 3,3-diaminobenzidine and counterstained with hematoxylin. Each photograph of the stained sections was scanned using a light microscope.

### 2.5. Immunofluorescence Staining

Immunofluorescence (IF) staining for a fluorescence microscope and GMCs were stained with anti-IL-6 antibody (mouse polyclonal antibody; 1 : 200 dilution; Cell Signaling Technology; USA), anti-COV IV (rabbit polyclonal antibody; 1 : 200 dilution; Abcam; UK), and anti-PanKbu (mouse polyclonal antibody; 1 : 200 dilution; Hangzhou Jing Jie biological Co., Ltd; China). Cy3/FITC immunofluorescence dye-conjugated secondary antibody (1 : 200 dilution; Biosynthesis Biotech; China) was incubated for 1 h at room temperature in the dark. The nucleus was labeled with DAPI, and images were taken with a fluorescence microscope (Leica, Germany).

### 2.6. Cell Culture and Treatment

Conditionally immortalized mouse glomerular mesangial cells (GMCs, SV-40 MES 13) were obtained from the China Centre for Type Culture Collection and cultured in Dulbecco's modified Eagle's medium (Gibco, Waltham, MA, USA) containing 5.6 mM glucose and 10% fetal bovine serum (Gibco) at 37°C and 5% CO_2_. GMCs were exposed to normal glucose (5.5 mM) as normal control (NC), HG (30 mM), NaB (1 mM, Sigma-Aldrich, USA), and A485 (10 *μ*M, Selleck, USA) for 24 h.

### 2.7. Western Blotting

Total proteins of kidney tissue and GMCs were extracted with extraction buffer (RIPA). Nuclear proteins were extracted with the Nucleoprotein Extraction Kit protocol (Shanghai Sangon Biotech, China). Proteins were boiled in Sample Buffer and electrophoresed on 12% Bis-Tris Gel polyacrylamide gels. Proteins were transferred to a PVDF (Millipore) membrane, and nonspecific bindings were inhibited by incubation in 5% skim milk. Immunoblotting was performed using anti-IL-6 (mouse polyclonal antibody; 1 : 500 dilution; Santa Cruz Biotechnology; USA), anti-TGF-*β* (mouse polyclonal antibody; 1 : 500 dilution; Santa Cruz Biotechnology; USA), anti-MCP-1 (rabbit polyclonal antibody; 1 : 1000 dilution; Biyuntian Institute of Biotechnology; China), anti-PanKbu (mouse polyclonal antibody; 1 : 1000 dilution; Hangzhou Jing Jie biological Co., Ltd; China), anti-H3K9bu (mouse polyclonal antibody; 1 : 1000 dilution; Hangzhou Jing Jie biological Co., Ltd; China), anti-H3 (mouse polyclonal antibody; 1 : 2000 dilution; Hangzhou Jing Jie biological Co., Ltd; China), and anti-GAPDH (mouse polyclonal antibody; 1 : 2000 dilution; Biyuntian Institute of Biotechnology; China) overnight at 4°C. The proteins were detected with the HRP chemiluminescence reagent (Millipore, USA), and images were captured with the UVP imaging system (Bio-Rad, USA). ImageJ software was used for the analysis of bands.

### 2.8. Real-Time PCR Analysis

Total RNAs of renal tissue and GMCs were extracted with TRIzol (Invitrogen, USA). The ReverTra Ace qPCR RT Master Mix (FSQ-201, TOYOBO) was used for reverse transcription reaction, and the QuantiNova SYBR Green PCR Kit (QIAGEN, German) was used for qRT-PCR. The qRT-PCR was performed with the Analytik Jena qTOWER 3 G real-time PCR system (JENA, Germany) according to the manufacturer's instructions. Primers used in this study were shown below: MCP-1: 5′TTAAAAACCTGGATCGGAACCAA′, 5′GCATTAGCTTCAGATTTACGGGT3′; IL-6: 5′TAGTCCTTCCTACCCCAATTTCC3′, 5′ATCTTTTGGGGTCCGTCAACT3′; TGF-*β*: 5′CTCCCGTGGCTTCTAGTGC3′, 5′GCCTTAGTTTGGACAGGATCTG3′; and Fn: 5′ATGTGGACCCCTCCTGATAGT3′, 5′GCCCAGTGATTTCAGCAAAGG3′. GAPDH was used as an internal reference gene to normalize target gene expression. All the samples were used in triplicates. The 2−*ΔΔ*Ct method was used to calculate the relative gene expression in comparison with the reference gene.

### 2.9. Statistics

Data are expressed as the means ± standard deviation (SD). Student's *t*-test was employed for comparisons between two groups. Differences were evaluated using GraphPad Prism9. *P* < 0.05 was considered statistically significant. The statistical significance was ^∗^*P* < 0.05,  ^∗∗^0.001 < *P* < 0.01,  ^∗∗∗^*P* < 0.001, and^∗∗∗∗^*P* < 0.0001.

## 3. Results

### 3.1. NaB Ameliorates the Disorder of Glucose and Lipid Metabolism in DKD Mice

**Figure 1:**
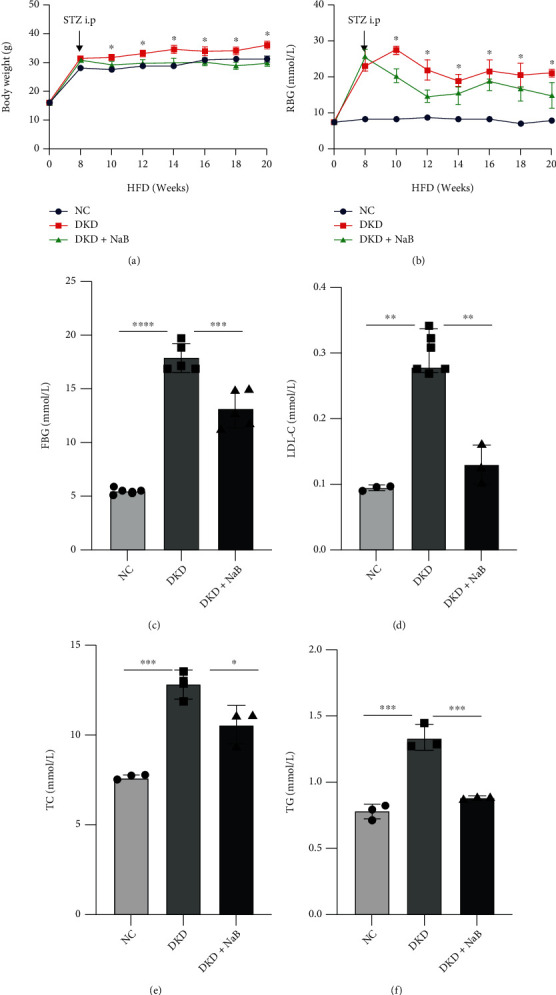
NaB ameliorates glucose and lipid metabolism disorder in DKD mice. Mice were subjected to a high-fat diet (HFD) for 8 weeks, intraperitoneally (i.p.) injected with STZ, and then treated with sodium butyrate (NaB), for 12 weeks. Body weight (BW) (a) and random blood glucose (RBG) (b) were measured every 2 weeks; fasting blood glucose (FBG) (c), low-density lipoprotein-cholesterol (LDL-C) (d), total cholesterol (TC) (e), and total glyceride (TG) (f) values were measured at the 20th week of the experiment before sacrifice. Values are presented as the mean ± SD. ^∗^*P* < 0.05,  ^∗∗^0.001 < *P* < 0.01,  ^∗∗∗^*P* < 0.001, and^∗∗∗∗^*P* < 0.0001.

To examine the effects of exogenous NaB on glycolipid metabolism in a nongenetic rodent model of DKD mice, body weight (BW, Figure 1(a)) and RBG (Figure 1(b)) levels were assessed from 8 to 20 weeks. After 8 weeks, compared with the NC group, significant changes in BW and RBG levels in DKD mice were noted. This pattern was also seen for FBG (Figure 1(c)), LDL-C, TC, and TG (Figures 1(d)–1(f)), suggesting that DKD models were successfully achieved. Next, we found that intraperitoneal injections of NaB for 12 weeks did have significant effects on BW, RGB, and the serum lipid spectrum in DKD mice. Collectively, our observations indicated that NaB did significantly ameliorate obesity and disorders of glucose and lipid metabolism.

### 3.2. NaB Alleviates Inflammatory and Fibrotic Injury in DKD Mice and High Glucose-Induced GMCs

**Figure 2:**
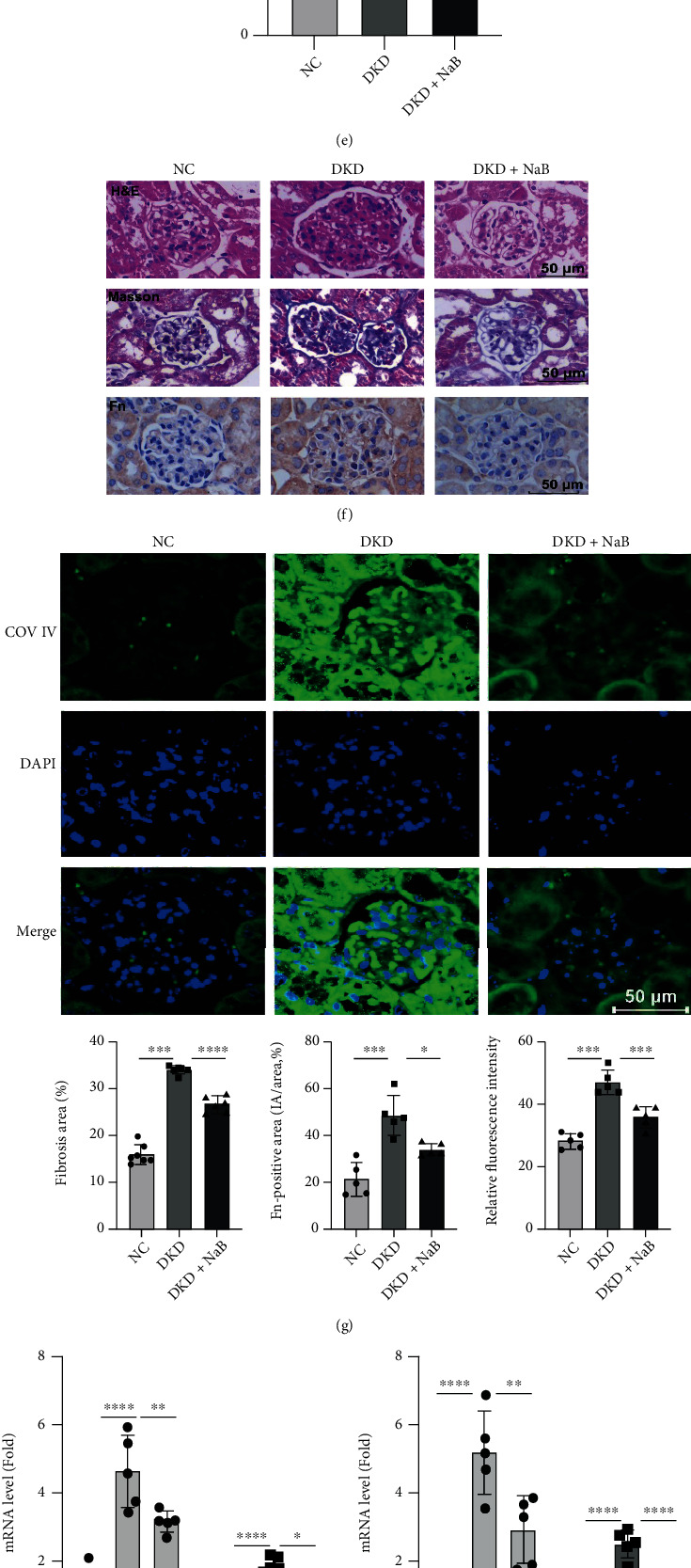
NaB alleviates inflammatory and fibrotic injury in DKD mice and high glucose-induced GMCs. Urine ACR (a), blood urea nitrogen (BUN) (b), blood crea (c), serum IL-6 (d), and serum MCP-1 (e) were assayed at the 20th week of the experiment; H&E and Masson staining of mice in each group (×400) and immunohistochemistry were used to detect the expression of Fn in mouse kidney of each group (×400) (f). The expression of mainly the contents of collagen type IV (COL IV) in kidneys of each group was detected by immunofluorescence (×400) (g); qRT-PCR of Fn, TGF-*β*, IL-6, and MCP-1 in kidney tissue after NaB treatment (h, i); Western blotting-based assays for the expression of TGF-*β* and MCP-1 in GMCs after NaB intervention (j); GMCs were stimulated with 30 mM high glucose in the presence of the indicated concentration of NaB for 24 h. MCP-1 and IL-6 in the cell culture supernatant were evaluated by the kit (k, l). Values are presented as the mean ± SD. ^∗^*P* < 0.05,  ^∗∗^0.001 < *P* < 0.01,  ^∗∗∗^*P* < 0.001, and^∗∗∗∗^*P* < 0.0001.

To assess whether NaB alleviates DKD *in vivo*, ACR, an important feature of DKD, was measured after the intervention. We found that NaB treatment resulted in lowering the levels of urine ACR (Figure 2(a)) and in lowering serum urea (Figure 2(b)) and creatinine (Figure 2(c)) levels, markers of the severity of renal dysfunction in DKD. NaB treatment also reduced serum levels of IL-6 (Figure 2(d)) and MCP-1 (Figure 2(e)), a sign that DKD is in a chronic, low-grade inflammatory state. Histopathological examination of renal tissues (Figure 2(f)) by H&E and Masson's trichrome revealed that mesangial expansion, the glomerular tuft, and the accumulation of collagen were substantially elevated in the DKD group when compared to the NC group. Notably, these histomorphometric changes were significantly attenuated by treatment with NaB. Immunohistochemical staining showed that NaB inhibited Fn expression in DKD (Figure 2(f)). Immunofluorescence staining revealed that NaB remarkably reduced glomerular collagen fibril IV deposition (COV IV) (Figure 2(g)); NaB effectively downregulated TGF-*β*, Fn, IL-6, and MCP-1 expression in DKD kidney tissues by qRT-PCR (Figures 2(h) and 2(i)); NaB downregulated the protein expression of IL-6 and TGF-*β* in cells induced by high glucose by Western blotting (Figure 2(j)). NaB inhibits MCP-1 and IL-6 release from GMCs induced by high glucose (Figures 2(k) and 2(l)). These results indicate that NaB inhibits renal inflammation and fibrosis gene expression and has a protective effect on DKD mice and high glucose-induced GMCs.

### 3.3. NaB Induces Histone Kbu in DKD Mice and GMCs

**Figure 3:**
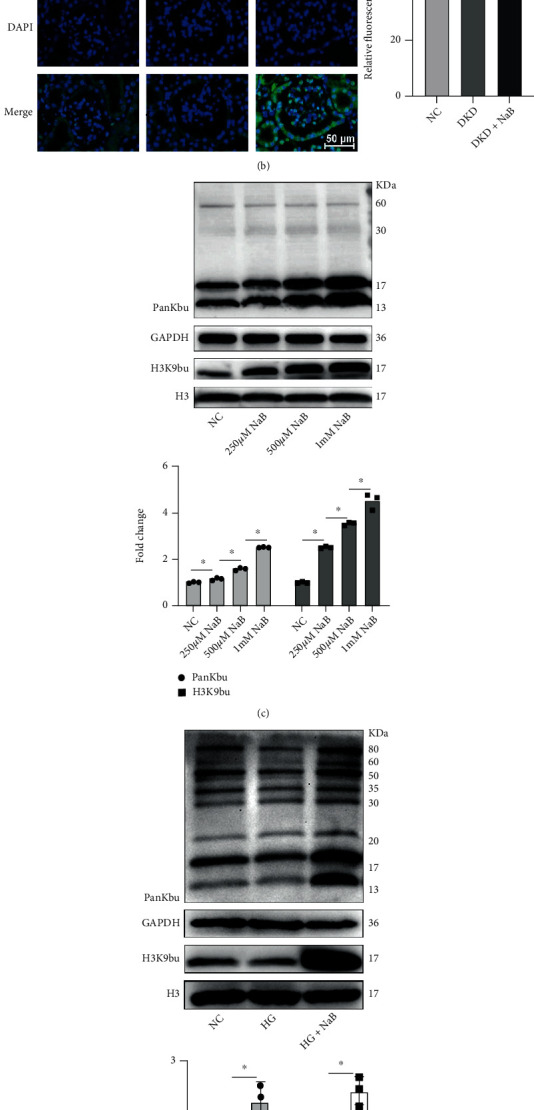
NaB induces histone Kbu in DKD kidney and renal mesangial cells. Immunohistochemistry (400x)-based assays for the expression of PanKbu and H3K9bu in DKD kidney tissue after NaB treatment (a). The expression of PanKbu in kidneys of each group was detected by immunofluorescence (×400) (b). The expression of PanKbu and H3K9bu after different concentrations of NaB intervened in GMCs (c). The expression of PanKbu and H3K9bu in GMCs of each group (d). The expression of H3K9bu in the nucleus in each group of GMCs (e). Values are presented as the mean ± SD. ^∗^*P* < 0.05,  ^∗∗^0.001 < *P* < 0.01,  ^∗∗∗^*P* < 0.001, and^∗∗∗∗^*P* < 0.0001.

The expression of PanKbu, H3K9bu, and P300 was downregulated in the kidney of DKD mice by immunohistochemistry, but this was reversed by NaB treatment (Figure 3(a)); NaB-induced expression of PanKbu was found to be predominantly in the nucleus (Figure 3(b)). Our results showed that PanKbu and H3K9bu were induced by NaB in a dose-dependent manner (Figure 3(c)). These results suggest that histone Kbu levels are regulated by cellular NaB concentration. The NaB intervention group significantly induced protein upregulation of PanKbu and H3Kbu compared to the NC and HG groups (Figure 3(d)). Moreover, extraction of nuclear proteins revealed that H3K9bu modifications were obviously upregulated (Figure 3(e)). These results indicate that NaB upregulates histone Kbu and H3K9bu modification levels in diabetic kidney tissue and GMCs.

### 3.4. A485 Inhibits Histone Kbu and Reverses NaB-Mediated Anti-inflammatory and Anti-fibrotic Effects

**Figure 4:**
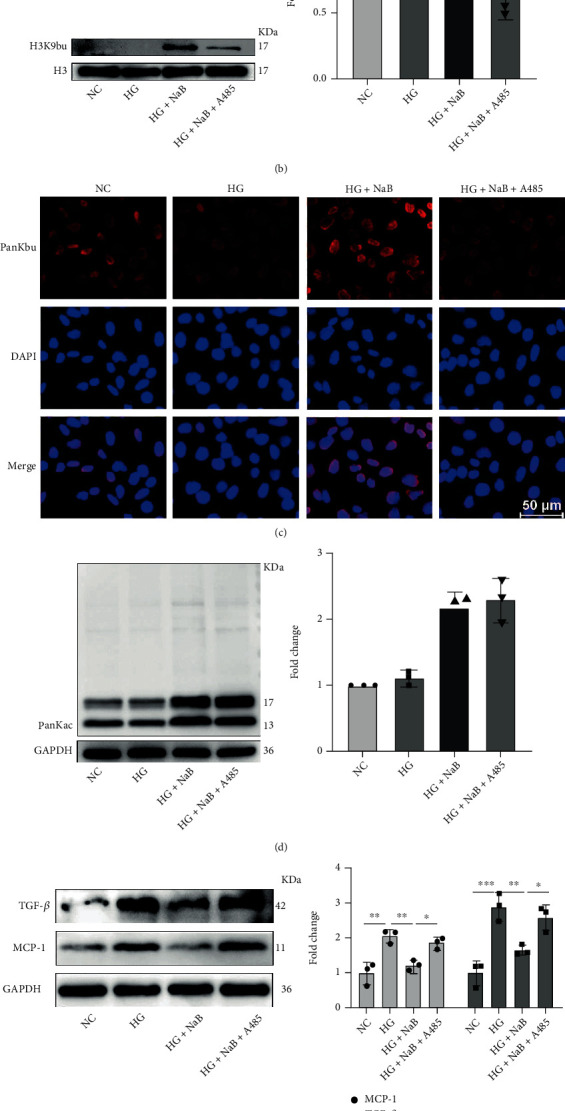
A485 inhibits NaB-mediated histone Kbu and reverses anti-inflammatory and antifibrotic effects. Effect of the P300 inhibitor A485 on the expression of PanKbu and H3K9bu by Western blotting (a). The expression of H3K9bu in the nucleus of GMCs in each group by Western blotting (b). The expression of PanKbu in GMCs of each group was detected by immunofluorescence (×200) (c). A485 had no significant effect on NaB-induced PanKac modification of GMCs (d). The NaB inhibition of TGF-*β* and MCP-1 was reversed by A485 (e). qRT-PCR was performed to detect Fn and TGF-*β* mRNA levels (f). The expression of IL-6 in cells of each group was detected by immunofluorescence (×200) (g). Values are presented as the mean ± SD. ^∗^*P* < 0.05,  ^∗∗^0.001 < *P* < 0.01,  ^∗∗∗^*P* < 0.001, and^∗∗∗∗^*P* < 0.0001.

To determine whether A485, an inhibitor of P300, inhibits histone Kbu *in vitro*, we firstly detected PanKbu and H3K9bu modification levels, and the results showed that A485 inhibited NaB-induced upregulation of PanKbu and H3K9bu (Figure 4(a)). H3K9bu modifications at the nuclear protein level are consistent with the total protein trend (Figure 4(b)). Furthermore, immunofluorescence showed that PanKbu and H3K9bu modification was mainly expressed in the nucleus, and A485 also inhibited NaB-induced histone Kbu (Figure 4(c)). A485 had no significant effect on NaB-induced pan-acetylation (PanKac) modification of GMCs (Figure 4(d). Western blotting confirmed that TGF-*β* and MCP-1 expression in GMCs was elevated in response to 30 mM glucose, revealing that the development of DKD is closely related to chronic inflammation and fibrosis. However, NaB-inhibited TGF-*β* and MCP-1 expression was abolished by A485 (Figure 4(e)). Furthermore, the results of qRT-PCR also demonstrated that A485 enabled the reversal of downregulation of Fn and TGF-*β* with NaB (Figure 4(f)). Consistent with Western blotting and qRT-PCR, immunofluorescence results indicated that A485 reversed the NaB-mediated inhibitory effect on IL-6 expression (Figure 4(g)). These results suggest that NaB may inhibit inflammation and fibrosis gene expression and ameliorate DKD through the histone Kbu pathway.

## 4. Discussion

It is crucial to suppress the expression of inflammatory and fibrotic genes to prevent and treat DKD. The latest research found that NaB inhibits oxidative stress and inflammatory gene expression and then ameliorates DKD via inhibition of histone deacetylases (HDAC) [12, 13]. Furthermore, NaB also alleviates glucolipid metabolism disorders by increasing glucagon-like peptide-1 (GLP-1) and insulin secretion [14]. In the present study, we found that intraperitoneal injection of NaB decreased BW, RGB, and FBG in DKD mice, as well as antagonized dyslipidemia, suggesting that NaB has a positive effect on maintaining homeostasis of glucolipid metabolism through a nongastrointestinal intervention. We speculate that NaB alleviates DKD glucolipid metabolism disorders by improving increasing GLP-1 and insulin secretion resistance as reported in the literature above. Here, our data found that elevated ACR, serum urea, and creatinine, markers of the severity of DKD, were decreased by NaB. In addition, mesangial expansion, the glomerular tuft, the accumulation of COV IV, and the expression of Fn in DKD renal tissues were remarkably attenuated by NaB. The inhibitory effects of NaB on oxidative stress and inflammation have been reported in GMCs induced by high glucose and lipopolysaccharide (LPS) [15]. Our study found that the protective effects of NaB were associated with the inhibition of IL-6 and MCP-1 in high glucose-induced GMCs. Taking into account all these recent studies and current results, exogenous NaB suppresses renal inflammation and fibrosis gene expression; NaB may be a potential therapeutic agent in the prevention and treatment of DKD.

However, the mechanism of NaB improving glucose and lipid metabolism and protecting DKD kidneys is still not fully understood. With the application of MS-based proteomics, novel histone lysine acylation has been successively discovered, such as lysine butyrylation (Kbu) [11], lysine *β*-hydroxybutyrylation (Kbhb) [16], and lysine crotonylation (Kcr) [17]; these modifications greatly enrich our understanding of epigenetics and histone code and provide a new strategy to explore the molecular mechanism of NaB. Histone Kbu induced by NaB [18] is specifically expressed in tumor [19], obesity [20], male spermatogenesis disorder [21], and other disease models. Whether NaB attenuates DKD and inhibits inflammation and fibrosis via histone Kbu has not been reported. This study shows that PanKbu, H3K9bu, and P300 were decreased in the kidney of DKD mice, suggesting that there is a correlation between the pathogenesis of DKD and endogenous butyrate; however, exogenous NaB induces the level of histone Kbu of GMCs in a dose-dependent manner, suggesting that the effect of NaB on DKD is related to histone Kbu. Recently, histone acetyltransferase P300 was found to be a coacyltransferase for novel HPTMs such as lysine acetylation (Kac), lysine butyrylation (Kbu), lysine *β*-hydroxybutyrylation (Kbhb), and lysine lactylation (Kla) [10, 22, 23]. Therefore, A485 might not be specific as an inhibitor of histone Kbu. While research methods are currently limited, most of the studies on novel HPTMs such as histone Kbhb have used A485 as an inhibitor of this modification [22]. Further research needs to explore specific modification enzymes or sites of Kbu and the effects on the expression of target genes.

Last but not least, the level of histone Kbu was significantly downregulated, and NaB-mediated anti-inflammatory and antifibrotic effects were blunted by A485. These indicate that NaB may suppress renal inflammatory and fibrotic gene expression and ameliorate DKD through P300-mediated histone Kbu. Although there is a lack of research to explore the molecular mechanism of histone Kbu, according to the existing research, histone Kbu may be synergistic or antagonistic with acetylation and methylation at the same or different modification sites. It forms a “histone code” through a cross-talk network and plays a key role in gene expression regulation and cell fate determination. Although the present study found no significant effect of A485 on NaB-induced acetylation levels in GMCs, whether there is an interaction between Kbu and Kac needs to be further confirmed by more investigations. Acetylation of histone H3K9 (H3K9ac) is involved in the pathogenesis of obesity in ob/ob mice and diabetes heart disease in db/db mice [24]. Recently, histone H3K9 *β*-hydroxybutyrylation (H3K9bhb) upregulates matrix metalloproteinase-2 (MMP-2) to antagonize glomerulosclerosis in diabetic rat [25]; H3K9bhb ameliorates aortic endothelial injury by promoting the generation of vascular endothelial growth factor (VEGF) in diabetic rats [26]. The above studies suggest that histone Kac, Kbhb, and Kbu may coexist or cross-talk at the H3K9 site, which may have synergistic or antagonistic effects on related gene expression. In this study, the histone H3K9bu was significantly upregulated by NaB, and the levels were downregulated after intervention with A485, which indicates that H3K9bu may be an important molecular target for NaB to alleviate kidney injury, and subsequent research needs to further explore more specific enzymes or modification sites by multiomics analysis.

Taken together, the present study reveals the molecular mechanism of butyrate from a new perspective, demonstrates that NaB may inhibit inflammation and fibrosis gene expression and ameliorate DKD through histone Kbu, and provides a basis for the future study or application of NaB, suggesting that novel histone modification may be a new target for the prevention and treatment of DKD.

## Data Availability

The data used to support the findings of this study are included within the article.
